# A new spin on chemotaxonomy: Using non‐proteogenic amino acids as a test case

**DOI:** 10.1002/aps3.70006

**Published:** 2025-04-14

**Authors:** Makenzie Gibson, William Thives Santos, Alan R. Oyler, Lucas Busta, Craig A. Schenck

**Affiliations:** ^1^ Interdisciplinary Plant Group, Department of Biochemistry University of Missouri Columbia Missouri USA; ^2^ Department of Chemistry and Biochemistry University of Minnesota Duluth Duluth Minnesota USA

**Keywords:** chemosystematics, evolution, metabolite, non‐proteogenic amino acid, phylogeny

## Abstract

**Premise:**

Specialized metabolites serve various roles for plants and humans. Unlike core metabolites, specialized metabolites are restricted to certain plant lineages; thus, in addition to their ecological functions, specialized metabolites can serve as diagnostic markers of plant lineages.

**Methods:**

We investigated the phylogenetic distribution of plant metabolites using non‐proteogenic amino acids (NPAA). Species–NPAA associations for eight NPAAs were identified from the existing literature and placed within a phylogenetic context using R packages and the Interactive Tree of Life. To confirm and extend the literature‐based NPAA distribution, we selected azetidine‐2‐carboxylic acid (Aze) and screened over 70 diverse plants using gas chromatography–mass spectrometry (GC‐MS).

**Results:**

Literature searches identified 163 NPAA‐relevant articles, which were manually inspected to identify 822 species–NPAA associations. NPAAs were mapped at the order and genus level, revealing that some NPAAs are restricted to single orders, whereas others are present across divergent taxa. The observed distribution of Aze across plants and ancestral state reconstruction suggests a convergent evolutionary history.

**Discussion:**

Although reliance on chemotaxonomy has decreased in recent years, there is still value in placing metabolites within a phylogenetic context to understand the evolutionary processes of plant chemical diversification. This approach can be applied to metabolites present in any organism and compared at a range of taxonomic levels.

Plants are remarkable for their chemical diversity. These metabolites enable plants to interact with their environment and provide various services to humans including medicines (Li and Weng, 2017; Erb and Kliebenstein, 2020; Weng et al., 2021). Roughly one‐third of medicines are derived from plants, and much of the global population relies directly on plants as medicinal sources (McChesney et al., 2007; Newman and Cragg, 2020). Estimates of total plant chemical diversity vary from 200,000 to over 1 million distinct metabolites (Dixon and Strack, 2003; Rai et al., 2017; Alseekh and Fernie, 2018); however, chemical diversity is difficult to estimate and likely much greater than these estimates, as many plants remain uninvestigated and some metabolites are beyond current analytical and detection methods. This underscores the relatively untapped potential of plants as a source for new chemistries that can be particularly useful in human health and disease treatment.

Core metabolites (also known as primary metabolites) are ubiquitous, whereas some specialized metabolites are produced in only a narrow range of plants or a single species (e.g., morphine; Beaudoin and Facchini, 2014), are produced by related species within the same family or order (e.g., glucosinolates; Blažević et al., 2020), or are accumulated in unrelated plants (e.g., caffeine and acylsugars; Huang et al., 2016; Kruse et al., 2022; Vendemiatti et al., 2024). Thus, lineage‐restricted metabolites can serve as diagnostic traits in plant taxonomy.

The field of chemotaxonomy combines chemistry and systematics (Alston and Turner, 1963; Gibbs, 1974; Reynolds, 2007), and early literature in the field focused on building taxonomic trees based on abundant plant metabolites (Bate‐Smith, 1962). Chemotaxonomy was useful for some metabolites but was impractical for other metabolites given the numerous instances of convergent evolution of plant metabolic pathways (Pichersky and Lewinsohn, 2011). Although chemotaxonomy is still useful, it is now more practical to map metabolites onto existing high‐quality plant phylogenies based on sequence data, which are both abundant and of high quality (Qian and Jin, 2016; Leebens‐Mack et al., 2019; Zuntini et al., 2024). Mapping metabolic traits onto existing phylogenies provides an indication of how metabolic pathways emerge, enables hypotheses about convergent evolution, and provides new directions for the study of plant lineages and their metabolism.

In addition to the common amino acids used during protein biosynthesis, plants produce many non‐proteogenic amino acids (NPAAs), which have wide‐ranging functions including defense, storage, and signaling (Huang et al., 2011; Jander et al., 2020). Some NPAAs, such as *S*‐adenosylmethionine and ornithine, are widely distributed across plants and act as intermediates in core metabolism (Bell, 2003; Huang et al., 2011; Vranova et al., 2011); however, NPAAs are more typically restricted to certain lineages. Their roles in these plants remain elusive, but could be involved in defense, signaling, or energy storage, among other possible functions (Huang et al., 2011; Vranova et al., 2011). Many NPAAs are structural analogs of proteogenic amino acids and can be misincorporated during protein biosynthesis (Steele et al., 2021; Rodriguez‐Mias et al., 2022; Thives Santos et al., 2024), leading to proteotoxic effects and reduced growth (Lee et al., 2016; Thives Santos et al., 2024). Plants that produce NPAAs as a defense mechanism likely have evolved strategies to avoid autotoxicity, such as sequestration or highly specific protein biosynthetic machinery (Norris and Fowden, 1972). The metabolic pathways for NPAAs are diverse, yet many remain poorly understood. Some NPAAs are synthesized through modification of proteogenic amino acids (e.g., beta‐tyrosine; Yan et al., 2015), intermediates of biosynthetic pathways (e.g., *S*‐adenosylmethionine; Roje, 2006), or derived through pathways independent of the analogous amino acid (e.g., azetidine‐2‐carboxylic acid [Aze]; Leete et al., 1986). A greater understanding of the distribution of NPAAs can inform studies on biosynthetic pathways and the biochemical processes related to their mechanisms of action.

Here, we put a new spin on chemotaxonomy using NPAAs as a test class of metabolites, focusing on eight NPAAs that have been detected across multiple plants: Aze, canaline, canavanine, djenkolic acid, 5‐hydroxytryptophan, indospicine, meta‐tyrosine, and mimosine. Leveraging the abundant literature that exists for the detection of NPAAs in plants and high‐quality nucleotide‐based phylogenies, we used text mining and manual curation to establish a list of species–NPAA associations, which were then mapped onto existing plant phylogenies at varying taxonomic scales using R packages and the Interactive Tree of Life (iTOL; Letunic and Bork, 2024). To confirm and extend the species–NPAA associations from the literature, we extracted and detected Aze from more than 70 diverse plants and used ancestral state reconstruction to determine the evolutionary trajectory giving rise to the observed Aze distribution. Our results were consistent with the literature and suggest that Aze likely evolved through convergent evolution in divergent taxa. This study provides a template that can be applied to any (plant) metabolite, which can be used to understand the emergence of metabolic pathways.

## METHODS

### NPAA–plant associations in the existing literature

To understand the phylogenetic distribution of NPAAs, we mined the literature to identify species–NPAA associations. Because of their occurrence throughout the literature and their restricted phylogenetic distribution (Fowden, 1963; Bell, 1976, 2003; Huang et al., 2011), we focused on eight NPAAs that have been detected across multiple plants (i.e., Aze, canaline, canavanine, djenkolic acid, 5‐hydroxytryptophan, indospicine, meta‐tyrosine, and mimosine). NPAA searches were conducted in the spring of 2024 in SciFinder (Chemical Abstracts Service [CAS], Columbus, Ohio, USA) and PubMed (https://pubmed.ncbi.nlm.nih.gov/). Searches in SciFinder were conducted with the CAS registry number for the L configurations of the NPAAs using an English language filter, while searches in PubMed were conducted with compound names and names of analogs. A comprehensive list of 900 publications was identified using text mining searches that contained potential species–NPAA associations. This list was manually curated to (1) remove literature inadvertently captured in text mining searches and (2) remove duplicates, correct for usage of common names, and correct for nomenclatural changes, resulting in a confident list of plant–metabolite associations (Appendix S1). There are limitations to our large‐scale phylochemical mapping approach, for example, NPAA–species associations that do not exist can be inadvertently assigned because of discrepancies in names, nomenclatural changes, or use of common names, and known NPAA accumulation patterns may be missed because searches have only been conducted with English language filters.

#### Phylogenetic mapping of NPAAs

Plant–metabolite associations were mapped onto an existing plant megaphylogeny (Qian and Jin, 2016). We pruned the megaphylogeny to show only certain lineages using the *buildTree* function available at https://github.com/thebustalab/phylochemistry, then visualized trees using the R package ggtree (Yu et al., 2017). Metabolite associations were plotted alongside phylogenies using ggplot2 (Wickham, 2009) or iTOL (Letunic and Bork, 2024). For the species tree, a total of 789 species are represented on the tree, 395 from species with NPAA associations and 394 randomly selected species. Because NPAA accumulation is biased towards certain lineages, such as Fabaceae, we randomly selected additional plant species in R to provide a phylogeny that was more representative of flowering plants. For phylogenetic mapping, not all species present in the literature or analyzed for Aze content are present in the megaphylogeny (Qian and Jin, 2016), thus not all of the data are represented in the phylochemical maps. An additional source of error in phylochemical mapping approaches comes from the use of megaphylogenies that attempt to place thousands of taxonomically distant species. Although representations at large scales are accurate (e.g., order‐ and family‐level placements), genus‐level details within a family may differ from phylogenies that focus on individual genera or families. An example in our analysis is the placement of *Mimosa* outside of the Caesalpinioideae subfamily in the megaphylogeny (Qian and Jin, 2016). All code used in our analyses is available at https://github.com/thebustalab/npaa_distribution (see Data Availability Statement).

### Ancestral state reconstruction

To reconstruct Aze character states across our phylogenetic analysis, we performed ancestral state reconstructions using Mesquite version 3.81 (Maddison and Maddison, 2023). A genus‐level phylogeny was used representing species that were tested or identified in the literature with Aze data, and the Aze character state was scored as present if it was identified in either the lab or the literature and as absent if absent in both the lab and literature data. Aze character state reconstructions were performed using a parsimony analysis and were able to identify 10 character state changes.

### Aze detection from diverse plants

To confirm and extend the literature‐based approach, Aze was extracted, detected, and analyzed from various plant lineages. Plant material was obtained through multiple sources: fresh leaf tissue was obtained from the Missouri Botanical Garden (St. Louis, Missouri, USA) and the University of Missouri Botanical Garden (Columbia, Missouri, USA), and seeds were obtained from the United States Department of Agriculture (USDA) germplasm resource (see Appendix S2). In most cases, fresh leaf material was collected and flash frozen in liquid nitrogen, lyophilized for at least 48 h until completely dry, and stored until analysis. Dry plant material was placed in a 2‐, 15‐, or 50‐mL tube together with glass beads and ground to a fine powder using a bead mill (Spex Geno/Grinder; Cole‐Parmer, Vernon, Illinois, USA). Seeds were ground to a fine powder using a Perten Labmill 3310 (PerkinElmer, Waltham, Massachusetts, USA). Between 10 and 20 mg of finely ground plant material was added to 700 μL of water and used for extraction. Samples were incubated for 1 h at 50°C with vortexing every 15 min; 700 μL of chloroform was then added to the sample, followed by incubation for 1 h at 50°C with vortexing every 15 min. Extracts were spun at 3000 × *g* for 30 min at 4°C, then 650 μL of the aqueous layer was transferred to a glass vial and dried to completeness using a CentriVap SpeedVac System SPD140DDA (Thermo Scientific, Waltham, Massachusetts, USA). The dried extract was resuspended in 50 μL of a pyridine solution containing 15 mg/mL of methoxyamine HCl and incubated for 1 h at 50°C, then 50 mL of *N*‐methyl‐*N*‐(*tert*‐butyldimethylsilyl) trifluoroacetamide + 1% tert‐butyldimethylchlorosilane (MTBSTFA + 1% t‐BDMCS; Supelco, Sigma‐Aldrich, St. Louis, Missouri, USA) was added and incubated for 1 h at 50°C.

Derivatized samples were injected into an Agilent 5977C GC/MSD (Agilent Technologies, Santa Clara, California, USA) with authentic Aze standard (Sigma‐Aldrich). One microliter of sample was injected with a 10:1 split ratio onto a 60 m DB‐5ms column (Agilent Technologies). The initial oven temperature was 120°C, with an oven ramp rate of 6°C per minute until 300°C and a hold time of 8 min, and the inlet valve temperature was maintained at 280°C. The GC/MSD was operated in full scan mode from a mass‐to‐charge ratio (*m/z*) of 50–650. A limit of detection for Aze was determined through injection of a concentration gradient of Aze from 0–200 µM, yielding a limit of detection of 16.7 µM. Raw gas chromatography–mass spectrometry (GC‐MS) files have been deposited to Figshare under the project name “A new spin on chemotaxonomy: using non‐proteogenic amino acids as a test case” (see Data Availability Statement).

## RESULTS

### Identification of NPAA–species associations

**Figure 1:**
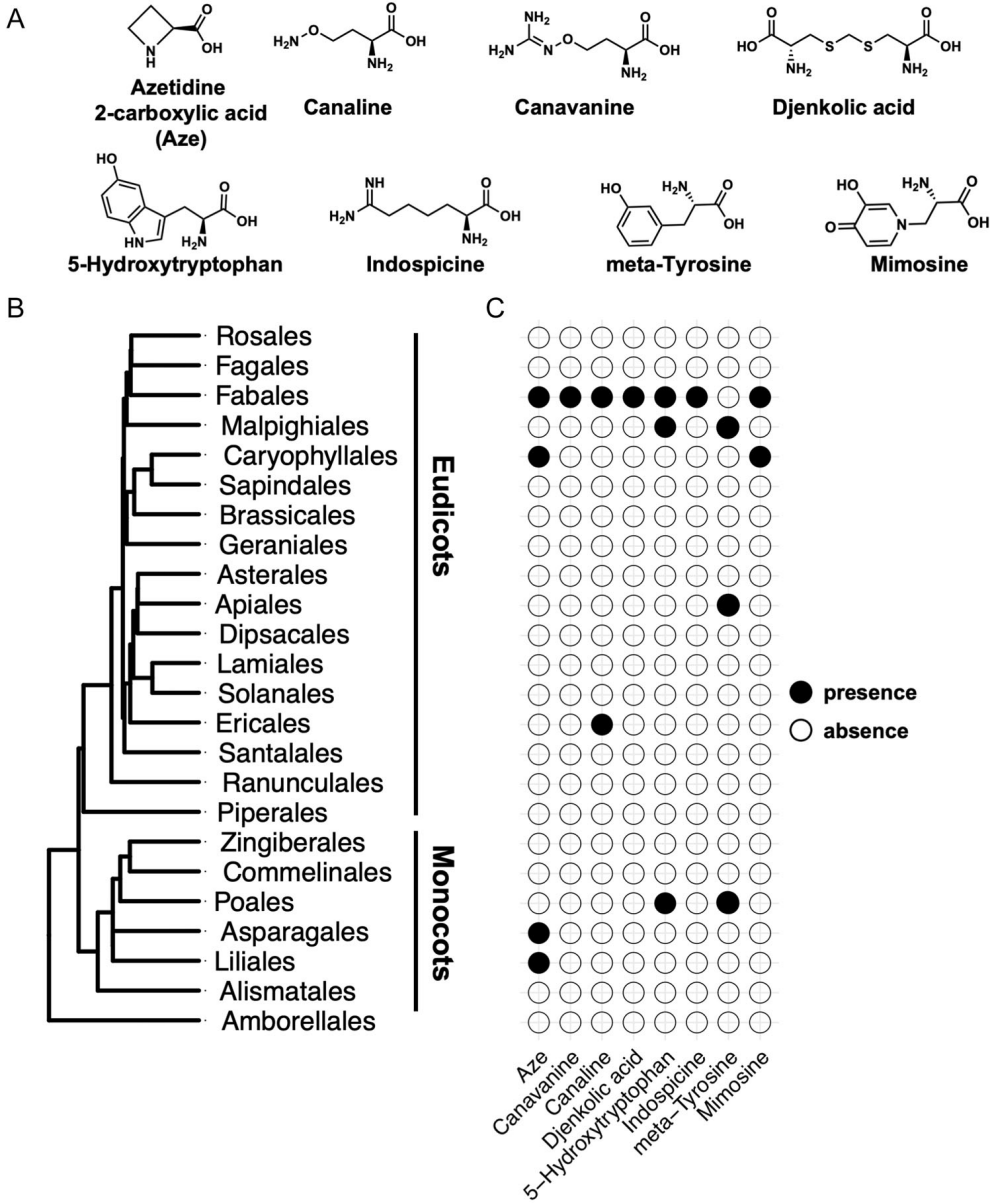
Order‐level distribution of NPAAs across plants. (A) Structures of the eight NPAAs that were a focus of this study. (B) Order‐level phylogeny of flowering plants. Twenty‐three orders were selected representing the diversity of flowering plants and a sister lineage (Amborellales). (C) Phylogenetic mapping of NPAA–species associations. Orders with species that contain a particular NPAA–species association in the literature are represented by a filled circle. Absence indicates that there was no literature to report an NPAA–species association, not necessarily lack of accumulation of that NPAA.

NPAAs were chosen as a framework for phylochemical mapping (Schenck and Busta, 2021; Busta et al., 2024) because there has been substantial interest in these compounds starting around the 1950s, and there is a large base of literature that contains NPAA detections across phylogenetically diverse plants (Grobbelaar et al., 1955; Fowden and Steward, 1957; Bell, 1976). We focused on eight structurally diverse NPAAs (i.e., Aze, canaline, canavanine, djenkolic acid, 5‐hydroxytryptophan, indospicine, meta‐tyrosine, and mimosine) (Figure 1A) with distinct mechanisms of action; some mimic proteogenic amino acids and are misincorporated during protein biosynthesis (Aze, meta‐tyrosine, and canavanine; Bertin et al., 2007; Zer et al., 2020; Thives Santos et al., 2024), some react with endogenous metabolites and cause adverse effects (canaline; Rosenthal, 1997), while others remain relatively unknown (indospicine, djenkolic acid). Literature searches identified more than 900 scientific articles with mentions of NPAAs, and our manual curation of these literature reports identified 822 verifiable NPAA–species associations in 163 unique articles (Appendix S1). A list of all sources used to identify species–metabolite associations can be found in Appendix S1. Canavanine was the most frequently detected NPAA in the literature with 572 reports, and canaline was the least detected with five associations identified (Appendix S1). This list of select NPAA–species associations was then used as the framework for mapping of the metabolic traits onto a phylogeny.

### NPAA distribution at the order level

To understand the distribution of the eight NPAAs at a global scale, order‐level phylogenies were constructed (Qian and Jin, 2016). Of the 64 orders recognized by the Angiosperm Phylogeny Group (Angiosperm Phylogeny Group et al., 2016), 23 orders were selected representing the diversity of flowering plants and used as a basis for mapping NPAAs using ggtree (Figure 1B) (Yu et al., 2017). Although NPAA–species associations were not identified for most of the orders on the phylogeny, the order‐level phylogeny provides a sense for the distribution of NPAAs across plants at a global scale. Aze, canaline, canavanine, djenkolic acid, 5‐hydroxytryptophan, indospicine, meta‐tyrosine, and mimosine were then plotted onto this phylogeny using ggplot2 (Wickham, 2009). In general, the NPAAs show a narrow distribution and are only found in a few orders (Figure 1C). The Fabaceae is well known for the accumulation of diverse NPAAs (Bell et al., 2008), and seven of the eight NPAAs were consistently found to associate with Fabales (Figure 1C). Some NPAAs were reported in only a single order and show a very narrow distribution (i.e., canavanine, djenkolic acid, and indospicine; Figure 1C). Despite canavanine being identified in over 400 species, all of these were within the Fabales order (Figure 1C). The other NPAAs were found in fewer species, but distributed across unrelated orders, such as mimosine in the Fabales and Caryophyllales and 5‐hydroxytryptophan in the Fabales, Malpighiales, and Poales (Figure 1C). The NPAAs that are reported in distinct orders could be examples of metabolites that have evolved through convergent evolution, but finer mapping and a deeper survey of species could provide more support for this hypothesis. It should be noted that the lack of a literature report of an NPAA does not indicate absence of the metabolite in that plant. Thus, although it appears as though NPAAs show limited distribution, this reflects sampling bias and lack of data.

### Species‐level NPAA distribution

**Figure 2:**
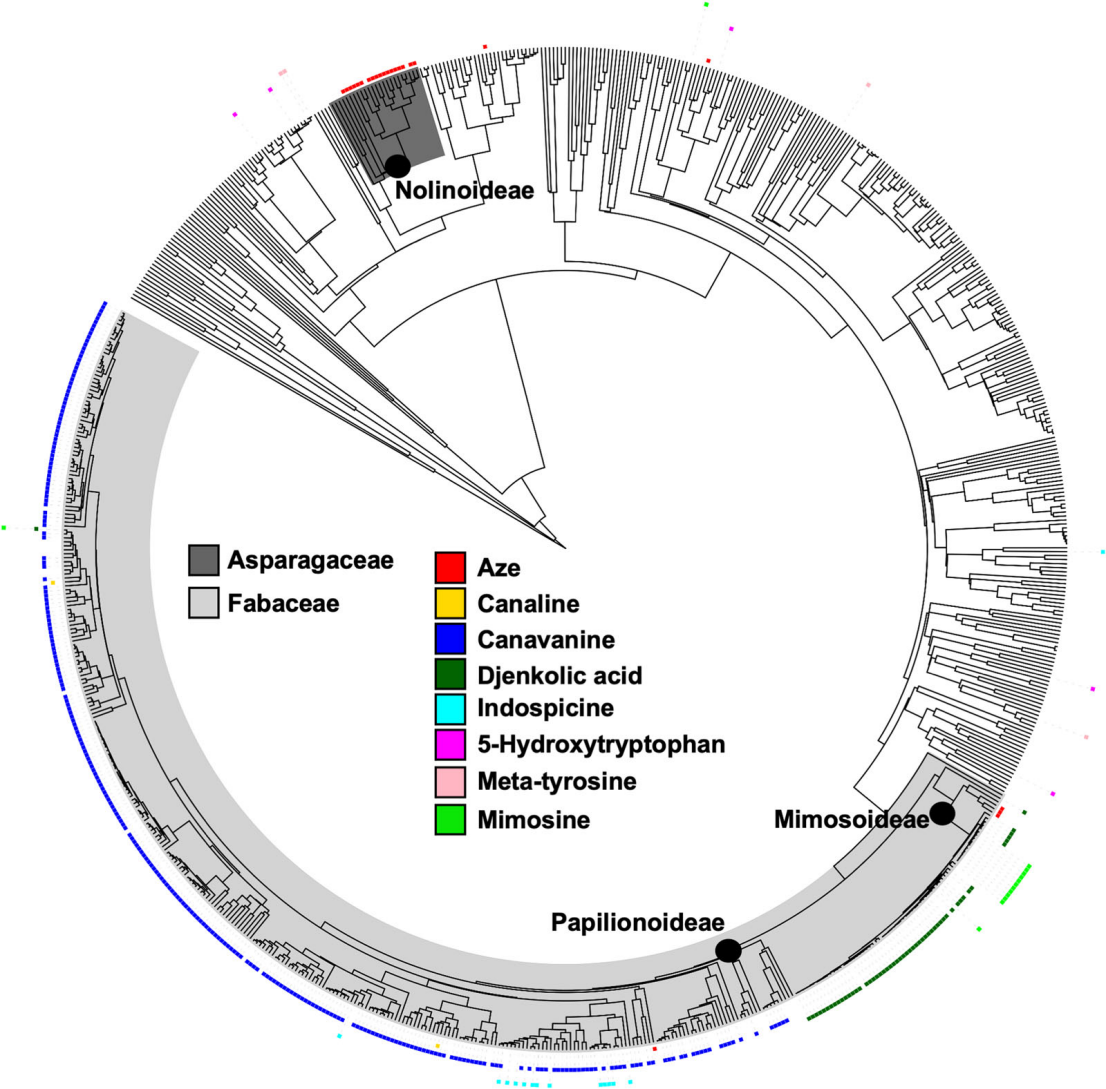
Species‐level distribution of NPAAs across plants. A species‐level phylogeny was built using species with NPAA associations and supplemented with 500 random species, resulting in a final tree containing 789 species. The eight NPAAs were mapped onto this tree. A filled box indicates that a particular NPAA has been reported in the literature for that species; species without boxes indicate lack of NPAA data. Plant names were removed for clarity; a full tree including the species names is provided in Appendix S3. Major plant clades are labeled on the tree, including families and subfamilies mentioned in the main text.

To gain a finer resolution of the distribution of the selected NPAAs across plants, we mapped distributions onto a species tree, using all species that had at least one NPAA detected and 500 randomly selected species to enrich the phylogenetic analysis. Not all the species with NPAA associations or the randomly selected species were present in the megaphylogeny we used to prepare our species‐level phylogenetic tree, thus only 789 species are represented on the species tree (Figure 2). Species names were removed from this tree for interpretability; however, a complete tree with species labels is provided in Appendix S3.

Canavanine has been detected in the most species, but all are restricted to the Fabales order (Figure 1). Within the Fabales, canavanine appears to be restricted to the Papilionoideae subfamily, which contains species such as alfalfa and common bean (Figure 2). Based on this phylogeny, it is likely that canavanine evolved in the common ancestor that has given rise to the modern‐day Papilionoideae subfamily and likely been retained in many, if not all, lineages. We found 75 djenkolic acid–species associations (Appendix S1). Djenkolic acid has only been detected in legume species (Figure 1C), all within the Mimosoideae subfamily and, apart from two exceptions, within the genus *Acacia* (Figure 2). Djenkolic acid likely emerged in the lineage that has given rise to *Acacia* and been retained in most, if not all, modern‐day *Acacia* lineages (Figure 2). Aze was identified 53 times in the literature and in four distinct orders (Figure 1, Appendix S1), and within those orders is restricted in its distribution to a few closely related legumes in the Caesalpinioideae subfamily and species within the Nolinoideae subfamily of the Asparagales order (Figure 2).

The other NPAAs have been reported in much fewer species. Mapping their distribution onto a species tree highlights the need for more sampling prior to making inferences about how these pathways may have evolved. Mimosine was identified 72 times in the literature, but only in 14 unique lineages, and all but one were within the legumes (Figure 2). All of these associations were found within the genera *Leucaena*, *Acacia*, and *Mimosa* (Figure 2), showing a limited distribution. Indospicine was identified 26 times within the literature, but only in 14 unique species, with all but one report within the Fabales. Within Fabales, indospicine is restricted to the genus *Indigofera* (Figure 2). 5‐hydroxytryptophan was detected in six species (Figure 2). Limited sampling limits further interpretation within a species context; however, 5‐hydroxytryptophan is widely distributed and present in three orders (Figure 1C). Meta‐tyrosine had six associations across three orders and is the only NPAA that was investigated that is not present within Fabales (Figure 2). Canaline was detected in three species, all but one of which were restricted to the legumes (Figure 2). Only a handful of species were reported to contain multiple NPAAs; for example, both mimosine and djenkolic acid have been reported in *Mimosa pudica* L., and canavanine and indospicine were both found within *Indigofera suffruticosa* Mill. (Figure 2, Appendix S1).

### Validation of the literature and extension of NPAA–plant associations

**Figure 3:**
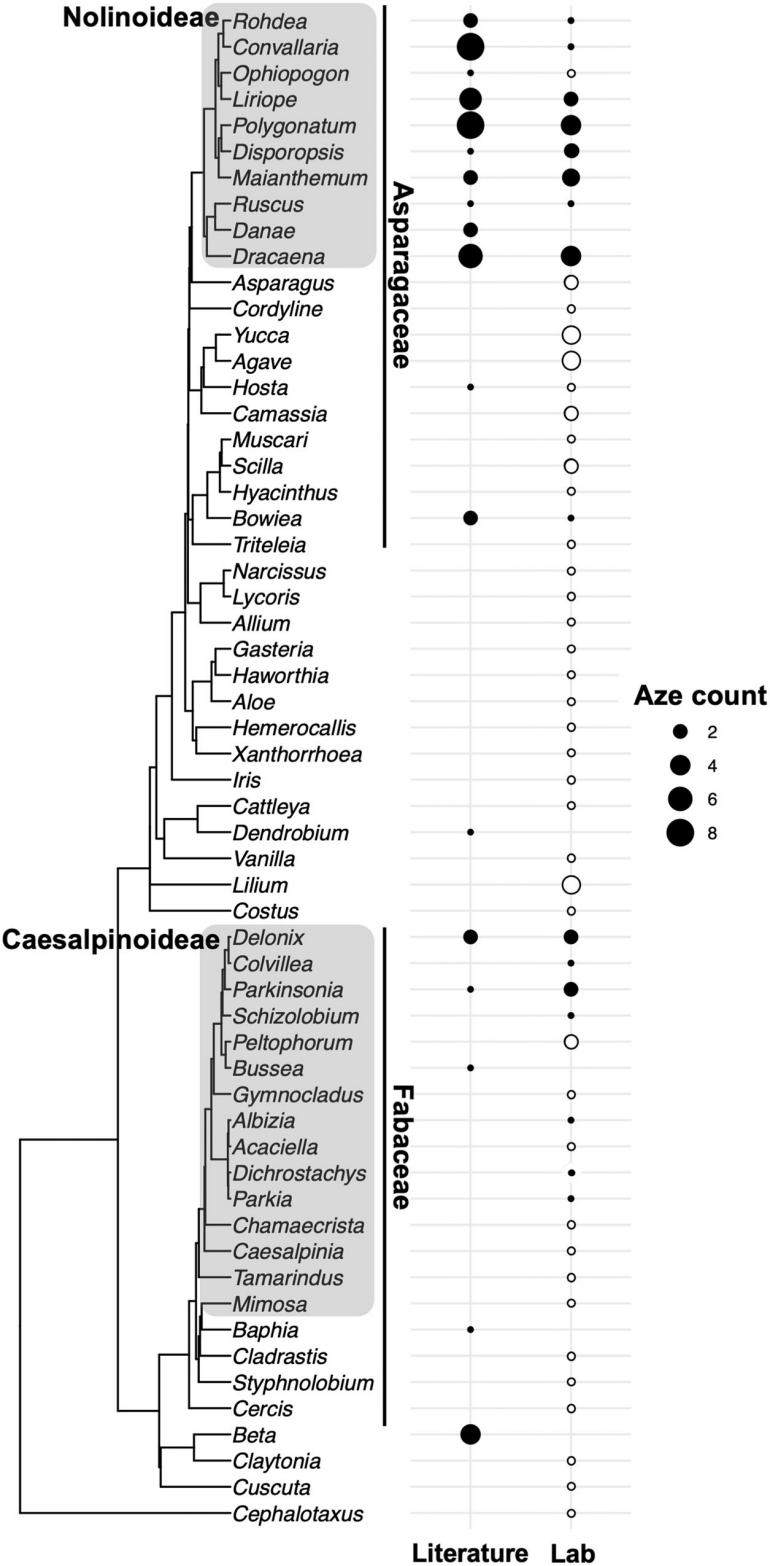
Confirmation and extension of literature‐based Aze distribution. Aze was mapped onto a genus‐level phylogeny primarily consisting of plants within the Fabales and Asparagales orders. For literature data (Literature), the size of the circle indicates the number of species and literature reports within each genus, with larger circles indicating more Aze–species associations. Seventy‐eight species were collected and analyzed for Aze using GC‐MS (Lab). The size of the circle indicates the number of species analyzed within each genus, with filled circles indicating the presence of Aze and unfilled circles indicating absence. Major plant clades are labeled next to the phylogeny.

To validate our literature‐based NPAA phylochemical mapping approach, we determined definitive species–Aze associations by metabolite extraction and detection using GC‐MS. Aze was first detected in *Convallaria majalis* L. (lily of the valley; Fowden, 1955) and, subsequently, in additional plants within the Asparagales and Fabales orders (Fowden and Steward, 1957; Sung and Fowden, 1969). We also used this opportunity to further refine Aze distribution by screening plants that are closely related to Aze accumulators. In total, we collected tissue from 78 species. Plants collected from botanical gardens were identified with family, genus, and species names and GPS coordinates to verify accurate collection; plants were also sourced from the USDA‐ARS Germplasm Resource Information Network (GRIN) and locally (Appendix S2). Aze was detected 53 times in the literature, and these associations were plotted onto a genus tree (Figure 3). Aze was mainly detected within Asparagales and Fabales (Figure 3), with reports of Aze also being detected from table beet within the genus *Beta* (Figure 3).

**Figure 4:**
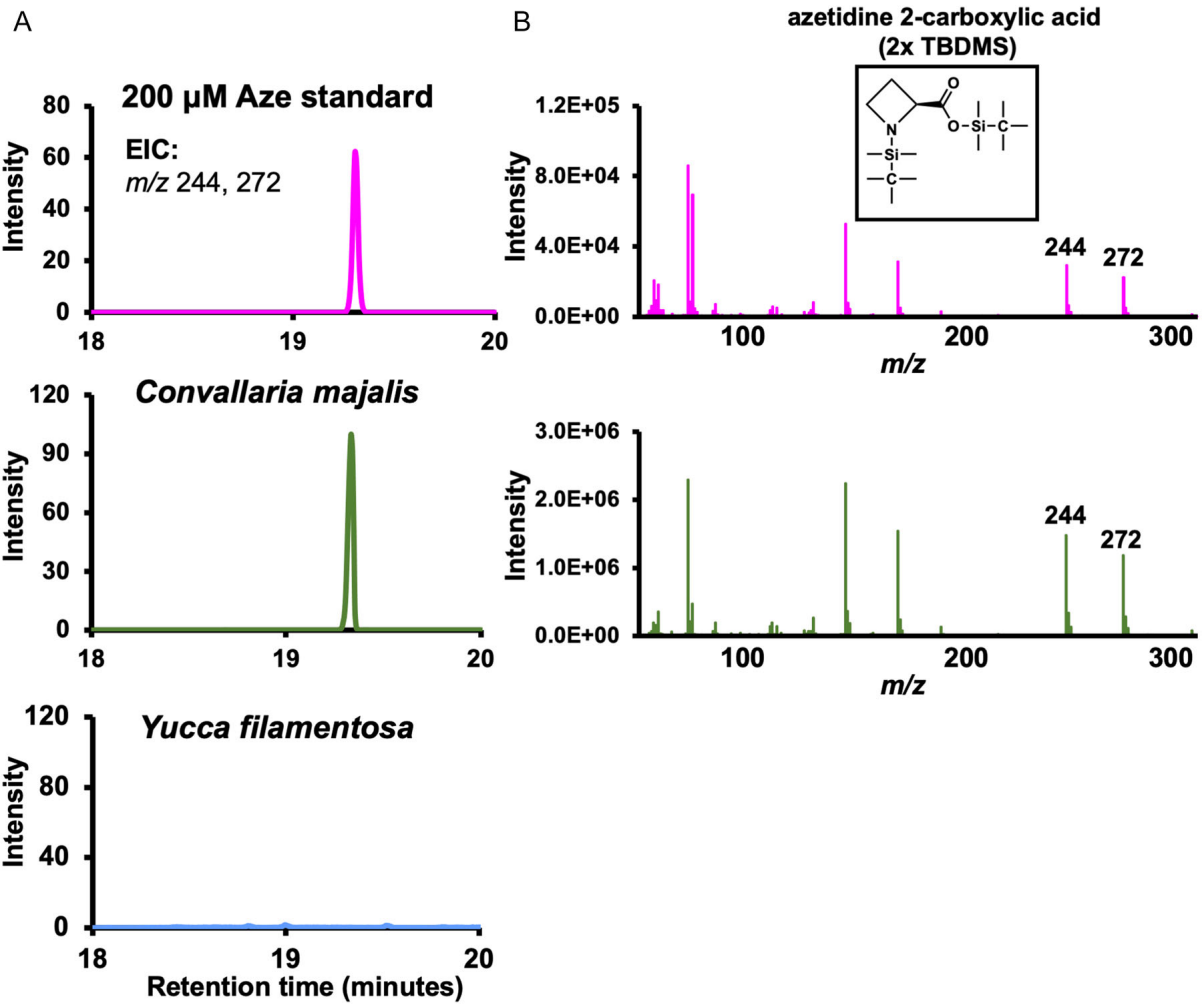
GC‐MS detection of Aze from plant extracts. Metabolite extractions were performed from lyophilized plant tissue, and dried extracts were derivatized with MTBSTFA and analyzed using GC‐MS. Authentic Aze standards (top row) were compared with plant extracts (middle and bottom rows). (A) Extracted ion chromatograms (EIC) for two ions (244 and 272) that are unique to Aze. The chromatograms show that the authentic Aze standard (pink) and an extract from *Convallaria majalis* (green) have a peak with the same retention time, which is absent in *Yucca filamentosa* (blue). (B) The mass spectra of Aze (pink) is nearly identical to Aze detected from *Convallaria majalis* (green). The inset at the top right shows derivatized Aze structure.

To isolate and detect Aze from diverse plants, we performed metabolite extractions primarily from leaf tissue, with other tissues used as indicated in Appendix S2, followed by derivatization and detection using GC‐MS (Figure 4). We developed methods using authentic Aze standards and used these to compare to plant extracts (Figure 4). Our Aze analyses mostly confirmed what has been reported in the literature (Figure 3), but also extend and refine the known distribution of Aze. Our analysis shows that Aze is narrowly distributed in both the Fabaceae and Asparagaceae (Figure 3). We initially hypothesized that Aze was only found within the Nolinoideae subfamily because a sister genus, *Asparagus*, does not accumulate Aze in two species tested (Figure 3). However, in both the literature and our analyses, Aze was detected in *Bowiea*, which is outside the Nolinoideae subfamily but within Asparagaceae (Figure 3). There was one case of Aze detection within the monocots, but outside Asparagaceae. Aze was detected in *Dendrobium* in the literature; however, we were unable to collect plant tissue from *Dendrobium* (Figure 3). *Dendrobium* could represent an interesting case of independent evolution of Aze, or a false positive. There are only a few instances of discrepancies between the literature and our Aze analysis. Using seeds, Fowden (1956) identified Aze–species associations for *Hosta*; however, we were unable to detect Aze from a single *Hosta* species (using leaf tissue). Additionally, Minakata et al. (1985) reported Aze in *Ophiopogon* (tissue type not reported); however, our analysis of a single *Ophiopogon* species (using leaf tissue) was unable to detect Aze (Figure 3).

Aze detection in legumes is narrowly reported in the literature (Figure 3), whereas our validation found that Aze is more widely distributed but only present within the Caesalpinioideae subfamily (Figure 3). There is one literature report for Aze in a *Baphia* species, which is outside the Caesalpinioideae but within Fabaceae (Figure 3). We were unable to collect and screen tissue for *Baphia*, but our screening of closely related legumes did not detect Aze in those lineages (Figure 3).

**Figure 5:**
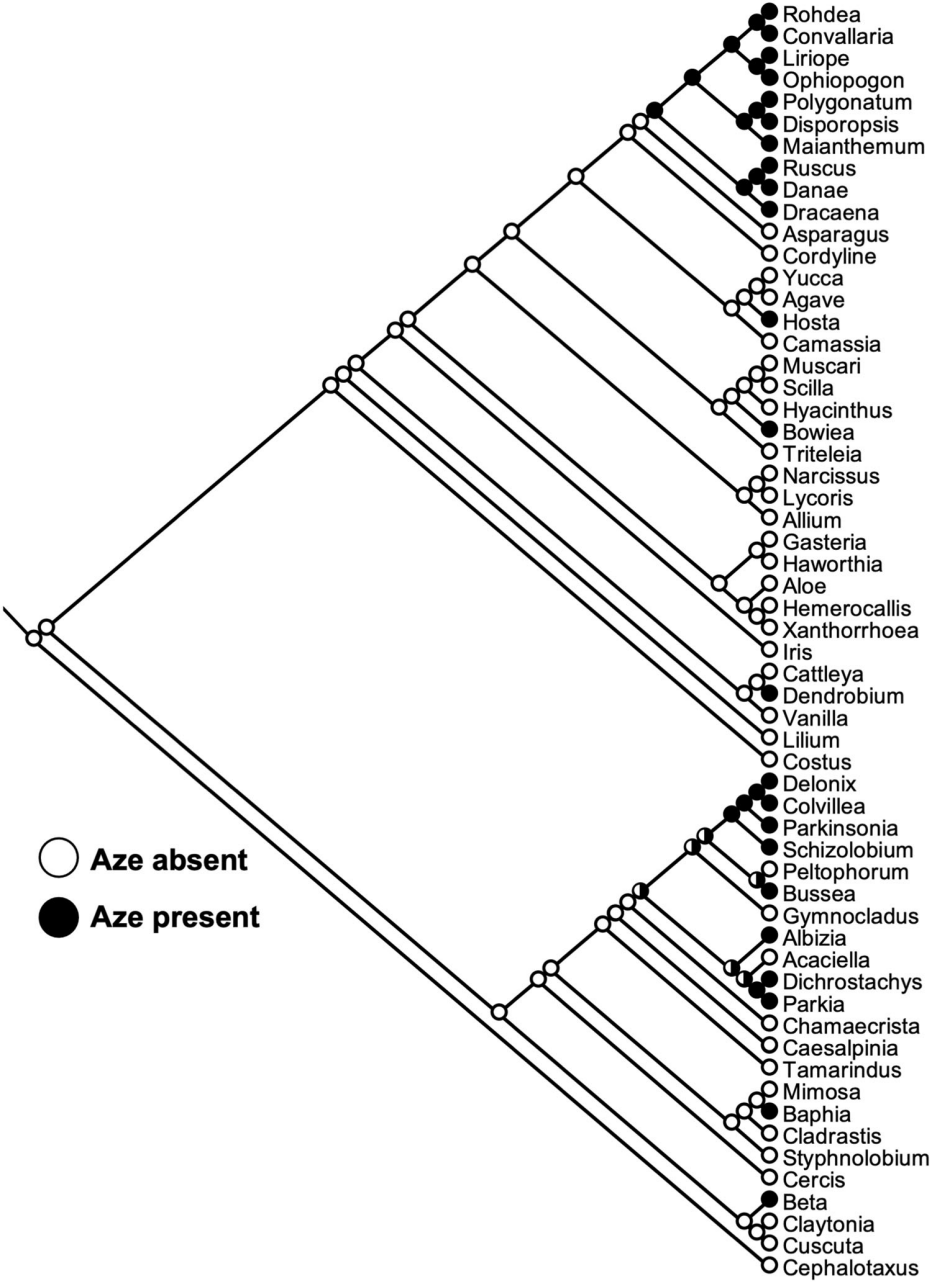
Reconstruction of Aze distribution across diverse taxa. Aze was mapped as a character state into a genus‐level phylogeny and was counted as present in a genus if it was detected in either the literature or the lab (see Figure 3). Filled circles indicate nodes that accumulate Aze, empty circles indicate nodes that do not accumulate Aze, and partially filled circles represent uncertainty in the ancestral state. Parsimony reconstruction was performed in Mesquite and shows 10 character state changes to explain the observed Aze distribution.

The observed distribution of Aze in divergent orders is suggestive of a pathway that has emerged independently in unrelated lineages. To test our hypothesis about the convergent evolution of Aze biosynthesis across diverse taxa, we performed ancestral character state reconstruction with parsimony analysis in Mesquite version 3.81 (Maddison and Maddison, 2023). Our analysis using combined Aze data from the literature and lab indicates that there are 10 character state changes across the observed distribution (Figure 5). Within monocots, Aze likely emerged four distinct times, including independent origins in *Dendrobium*, *Bowiea*, and *Hosta*, and another at the base of the subfamily Nolinoideae (Figure 5). Aze appears to have at least three origins in eudicots (Figure 5). Although the reconstruction is uncertain, it appears that Aze may also have been lost several times in the legumes (e.g., *Acaciella*, *Gymnocladus*, *Peltophorum*; Figure 5). The distribution of Aze across three distinct flowering plant orders (Fabales, Asparagales, and Caryophyllales) supports a convergent evolutionary hypothesis for the independent emergence of Aze biosynthesis, and future genomics and biochemical work could strengthen this hypothesis.

## DISCUSSION

In this study, we built phylochemical maps to depict the phylogenetic distributions of NPAAs across plants. Our analyses highlight the prevalence of NPAA production within legumes and additional orders. This approach can shed light on the emergence and potential loss of NPAA biosynthetic pathways and pinpoint species for additional biochemical and genomic studies.

### Increasing input data for phylochemical mapping

We chose eight NPAAs as a starting point, mostly because of their common occurrence in the literature. However, of the ~400,000 vascular plants (Christenhusz and Byng, 2016), only a small fraction has any literature evidence about the occurrence of the NPAAs that were the focus of this study. To increase the utility of phylochemical mapping, major improvements are needed, including increasing accessibility to large living collections of phylogenetically diverse plants, together with enhancements in metabolomics. The evolutionary hypotheses in our studies, and many others, could be strengthened by having data for plants at important phylogenetic positions; however, obtaining plant material for species of interest that are not model systems or crops remains a major bottleneck. Metabolomics methods could be improved for larger‐scale detection of many NPAAs in a single analysis, similar to large‐scale screening of metabolites across a diverse genus (Ernst et al., 2019). Many of the methods used to detect NPAAs have been optimized for a single metabolite, thus most papers report the association of one species to one metabolite or a few species to the same metabolite. Methods should be developed so that NPAAs can be identified and quantified at scale, similar to existing methods used to quantify the 20 proteogenic amino acids (Thomas et al., 2024; Zulfiqar et al., 2024). The simultaneous detection of many NPAAs from the same plant tissue will provide a holistic understanding of the types and distribution of NPAAs across plants. Additionally, approaches that combine large language models and machine learning algorithms could be developed that not only search for but assign metabolite–species associations with high accuracy and at large scales, thereby enhancing phylochemical mapping approaches (Busta et al., 2024). Another limitation is missing species in phylogenies that are considered comprehensive, resulting in phylochemical maps that lack metabolite data because species are absent from the phylogeny. Thus, continued improvements and representation in the plant tree of life are crucial for accurate phylochemical mapping and interpretation (Zuntini et al., 2024).

### Do most plants not produce NPAAs?

Our focused analysis of a few NPAAs suggests that most plant orders do not accumulate NPAAs (Figure 1); however, we have only mapped eight of the more than 200 distinct NPAA structures identified in plants (Huang et al., 2011; Vranova et al., 2011). Before drawing conclusions about the restriction of NPAAs to certain species and orders, a more global approach should be conducted. However, even from our focused NPAA analysis, it appears that some orders are better represented. For example, the Fabales, Malpighiales, Caryophyllales, and Poales all have multiple occurrences of structurally distinct NPAAs (Figure 1), and legumes are also well known for accumulation of structurally diverse NPAAs (Bell et al., 2008). This may suggest that these orders are predisposed to produce NPAAs, or that NPAAs may provide a selective advantage that is particularly important to these lineages. The correlation of NPAA accumulation and the geographic or environmental distribution of species that produce NPAAs may shed light on the function or mechanism of action of NPAAs. Most plants produce a suite of defense metabolites to target both generalist and specialist predators (Endara et al., 2023); thus, it is likely that plants accumulate multiple types of NPAAs and other specialized metabolites for diverse defensive functions. Combining the phylogenetic mapping of more NPAAs with genomic and biochemical studies could provide insight into the hypothesis that some lineages are predisposed for NPAA biosynthesis.

### Convergent evolution of some NPAA biosynthetic pathways

Convergent evolution is a common occurrence in plant specialized metabolism (Pichersky and Lewinsohn, 2011), with diverse metabolites arising independently in distinct lineages, including caffeine, betalains, and pyrrolizidine alkaloids (Reimann et al., 2004; Huang et al., 2016; Sheehan et al., 2020). Some NPAAs, such as Aze, are found in a few unrelated orders (Figures 1, 2, 3), and this distribution is highly suggestive of Aze biosynthesis independently emerging multiple times. Additionally, genus‐level phylogenies provide more resolution to when these pathways may have emerged (Figures 3 and 5), and in some instances, into lineages that seem to have lost these pathways (Figures 3 and 5). Similar patterns of loss and gain events have been observed in pyrrolizidine alkaloids, for which independent gain events have been followed by the subsequent loss of these pathways in some lineages (Livshultz et al., 2018). The lineages that have potentially lost Aze biosynthesis are equally interesting as those that have gained Aze biosynthesis and could provide insight into how and why metabolic pathways are lost. Metabolite distributions alone, however, only provide an indication of convergent evolution, and tracing the gain and loss of biosynthetic genes offers greater insight into pathway evolution. However, the Aze biosynthetic pathway in plants is not known.

### Phylochemical mapping to identify lineages for future investigations

The placement of metabolites onto a phylogenetic context provides an indication of how pathways emerged, and comparing biosynthetic pathways and the genes in distinct species may offer support for hypotheses about pathway evolution and loss events (Boachon et al., 2018; Lichman et al., 2020; Züst et al., 2020; Schenck et al., 2022). Phylochemical mapping can be broadly applied to metabolites distributed across any group of organisms, because it (1) leverages existing chemical data in the literature and (2) can be supplemented with chemical analysis with relative ease. Phylochemical mapping enables identification of plant lineages for follow‐up experiments, which can be more challenging to perform at scale. Metabolite distributions, together with complementary techniques such as genomics and biochemistry, can provide a full understanding of the evolution of metabolic pathways.

As an example of how phylochemical mapping can identify species for future studies, here we identified an interesting distribution of djenkolic acid and mimosine (Figure 2). Mimosine is narrowly distributed in two genera, *Mimosa* and *Leucaena*, whereas djenkolic acid is not reported in these genera, but is in all the surrounding genera (Figure 2). Thus, mimosine‐producing plants are embedded within the plants that produce djenkolic acid, and the distribution of these two NPAAs appears to be mutually exclusive. While it is possible that lack of data is partially responsible for this unique distribution of djenkolic acid and mimosine, it is also possible that metabolic changes in these species enable one NPAA to be produced and not the other. As a first follow‐up step, additional plants could be screened for the presence of djenkolic acid and mimosine. Then, comparative analyses using genomics and biochemistry of the legume lineages that produce djenkolic acid and mimosine could provide insight into how djenkolic acid and mimosine pathways evolved and whether these compounds are mutually exclusive. Phylochemical mapping approaches can be applied to metabolites distributed across organisms in any taxonomic grouping. Given the high‐quality genomes available across the tree of life, this approach can provide insight into how and why lineages produce specific metabolites. Ongoing advances in metabolomics, genomics, systematics, and high‐throughput literature scanning will enhance the effectiveness of phylochemical mapping and strengthen the conclusions drawn from chemotaxonomy.

## AUTHOR CONTRIBUTIONS

M.G. performed wet lab and computational experiments, analyzed data, created figures, and reviewed and revised the text. W.T.S. performed wet lab and computational experiments, analyzed data, created figures, and reviewed and revised the text. A.R.O. performed literature searches, compiled data, and edited the text. L.B. conceptualized the project, analyzed data, created figures, and edited the text. C.A.S. conceptualized the project, analyzed data, created figures, and wrote and edited the text. All authors approved the final version of the manuscript.

## Supporting information


**Appendix S1.** Metadata from the NPAA literature mining.

**Appendix S2.** Plant information used for Aze screening.

**Appendix S3.** Species‐level phylogenetic tree showing the distribution of NPAAs across plants, with the species labels included. A filled box indicates that a particular NPAA has been reported in the literature for that species; species without boxes indicate lack of NPAA data.

## Data Availability

All the relevant data and code are available in the Supporting Information or on our GitHub page (https://github.com/thebustalab/npaa_distribution). Raw GC‐MS files have been deposited to Figshare under the project name “A new spin on chemotaxonomy: using non‐proteogenic amino acids as a test case” (https://figshare.com/projects/A_new_spin_on_chemotaxonomy_using_non-proteogenic_amino_acids_as_a_test_case/230445).
