# Supplementary material for: A new spin on chemotaxonomy: Using non‐proteogenic amino acids as a test case
Source: Appl Plant Sci. 2025 Apr 14;13(4):e70006. doi: 10.1002/aps3.70006 (PMC12319704; doi:10.1002/aps3.70006)

DOI 10.1002/aps3.70006

APPENDIX S3. Species-level phylogenetic tree showing the distribution of NPAAs across plants, with the species labels included. A filled box indicates that a particular NPAA has been reported in the literature for that species; species without boxes indicate lack of NPAA data.

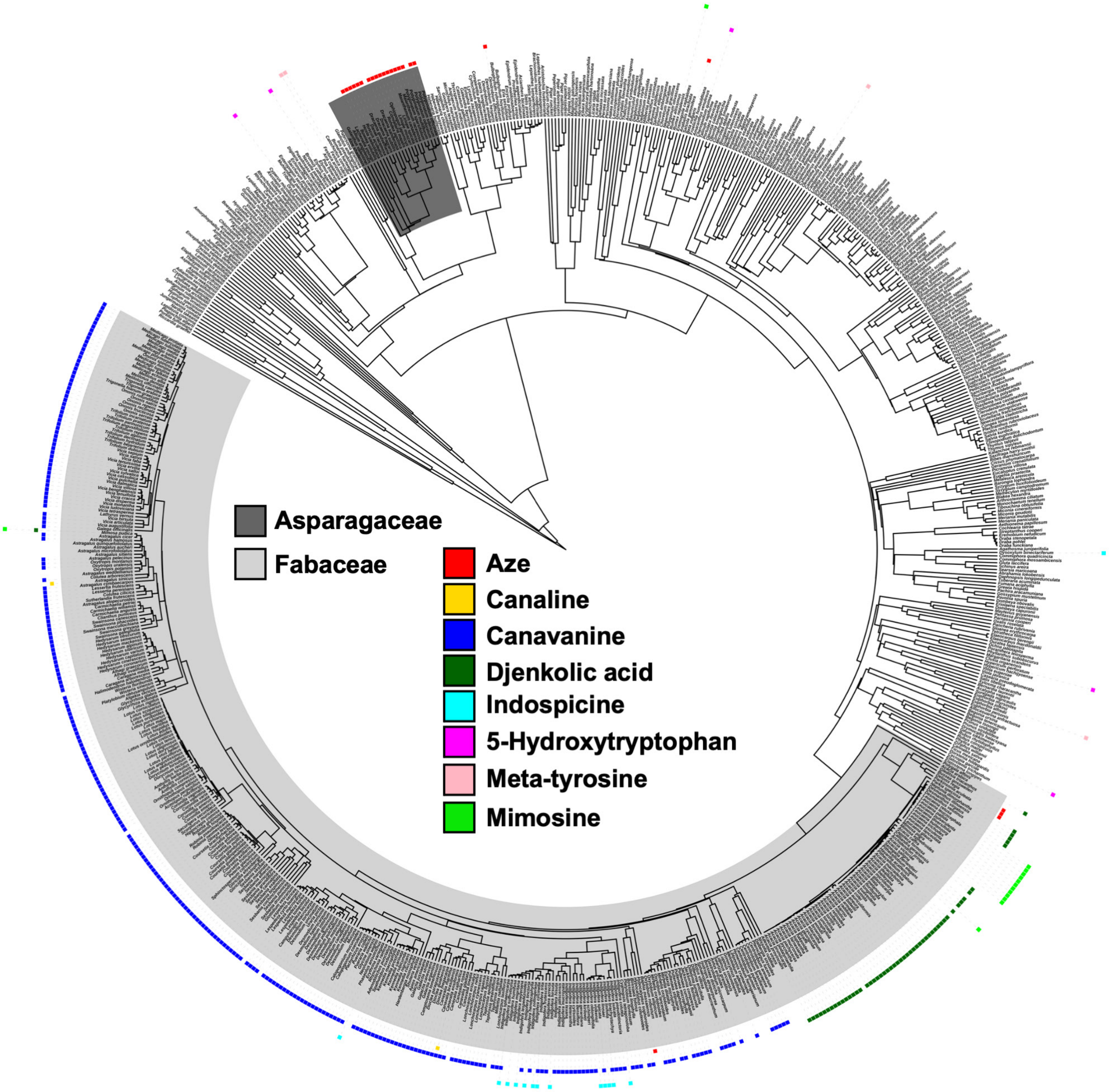

Supplement: Supplementary file 3 — Appendix S3. Species‐level phylogenetic tree showing the distribution of NPAAs across plants, with the species labels included. A filled box indicates that a particular NPAA has been reported in the literature for that species; species without boxes indicate lack of NPAA data. [file APS3-13-e70006-s003.pdf]
